# Error Consistency for Machine Learning Evaluation and Validation with Application to Biomedical Diagnostics

**DOI:** 10.3390/diagnostics13071315

**Published:** 2023-04-01

**Authors:** Jacob Levman, Bryan Ewenson, Joe Apaloo, Derek Berger, Pascal N. Tyrrell

**Affiliations:** 1 Department of Computer Science, St. Francis Xavier University, Antigonish, NS B2G 2W5, Canada; 2 Martinos Center for Biomedical Imaging, Massachusetts General Hospital, Department of Radiology, Harvard Medical School, Boston, MA 02129, USA; 3 Nova Scotia Health Authority, Halifax, NS B3H 1V7, Canada; 4 Department of Mathematics and Statistics, St. Francis Xavier University, Antigonish, NS B2G 2W5, Canada; 5 Department of Medical Imaging, Institute of Medical Science, University of Toronto, Toronto, ON M5T 1W7, Canada; 6 Department of Statistical Sciences, University of Toronto, Toronto, ON M5T 1W7, Canada

**Keywords:** classification, error consistency, supervised machine learning, validation

## Abstract

Supervised machine learning classification is the most common example of artificial intelligence (AI) in industry and in academic research. These technologies predict whether a series of measurements belong to one of multiple groups of examples on which the machine was previously trained. Prior to real-world deployment, all implementations need to be carefully evaluated with hold-out validation, where the algorithm is tested on different samples than it was provided for training, in order to ensure the generalizability and reliability of AI models. However, established methods for performing hold-out validation do not assess the consistency of the mistakes that the AI model makes during hold-out validation. Here, we show that in addition to standard methods, an enhanced technique for performing hold-out validation—that also assesses the consistency of the sample-wise mistakes made by the learning algorithm—can assist in the evaluation and design of reliable and predictable AI models. The technique can be applied to the validation of any supervised learning classification application, and we demonstrate the use of the technique on a variety of example biomedical diagnostic applications, which help illustrate the importance of producing reliable AI models. The validation software created is made publicly available, assisting anyone developing AI models for any supervised classification application in the creation of more reliable and predictable technologies.

## 1. Introduction

Supervised learning-based classification is a class of technologies tasked with predicting whether a set of measurements belong to a predefined group on which the algorithm was trained [1,2,3]. In the context of medical diagnostics, these supervised learning (SL) classification algorithms are regularly relied upon to predict whether a given patient belongs to a pathological group of interest; thus, many applications have been developed in which the trained SL model is responsible for the critical task of making a diagnosis [4,5,6]. In order to ensure that the SL model is of the highest quality, internal lab-based validation is typically performed prior to real-world deployment. A variety of validation procedures have been developed for the assessment of supervised machine learning (ML)-based classification algorithms [7,8]. Hold-out procedures are employed that divide the available data samples into distinct training and testing (and sometimes also validation) groups via techniques such as K-Fold cross validation [9] and Efron’s Bootstrap [10]. These validation techniques typically employ randomization in assigning samples to distinct training/testing groups, are repeated many times, and evaluative metrics are computed in each randomized run, such as overall accuracy (OA), the error rate, or the area under the receiver operating characteristic curve (AUC) [11]. The most common method for the validation-based evaluation of a ML algorithm is to assess the performance metric of interest across randomized validation runs. It is common for ML researchers to expect that this aggregated performance metric (such as the average accuracy) across validation runs will reflect ML performance in real-world deployment situations where the algorithm is exposed to entirely independent datasets. In situations where each of the ML models created across validation runs have learnt something quite different from each other, then the average performance may not be reflective of the real-world performance for any of the individual models created as part of validation. This can have a major impact on the real-world behavior of a SL model tasked with medical diagnostics, as the predictability of behavior and the reliability of predictions are critical to the development of clinically relied upon technologies.

When validation-created ML models disagree with each other, which one should be deployed for real-world operation? While this design question cannot be definitively answered at this time, in this manuscript we present an approach to expanding existing ML validation techniques to assess the degree to which ML models created across validation runs are consistent with each other, with a focus on whether the ML models agree on the sample-wise predictive mistakes the technology makes. Thus, we provide a method for assessing consistency between validation models. The approach presented focuses on the consistency of the distribution of samples on which the models make errors in an effort to better understand the shortcomings of the models created and their consistency with each other. Even in situations where all models created as part of validation yield the same (or almost the same) accuracies, it is possible for the various trained validation models to largely disagree on which samples they make their mistakes on. This can have a major impact on medical diagnostic applications, as the resultant technology will make erroneous diagnoses on different patients depending on which model created as part of validation is deployed clinically.

Reporting machine learning performance metrics, such as cross-validated overall accuracy [12], standard error [13] and confidence intervals [14], have long been common practice in evaluating applications of artificial intelligence. Previous related work is relatively limited and focuses on deep learning, unlike this analysis that focuses on traditional statistical learners. A large analysis was performed to account for variance in machine learning benchmarks for deep learning [15], in which a variety of sources of variation such as augmentation, data sampling, hyperparameter choices and parameter initialization were assessed for their impact on model performance. Assessments of both replicability (whether model performance is consistent across multiple attempts to train the learner with the same data) and reproducibility (whether model performance can be reproduced despite different real-world data) were addressed in a detailed study focused on deep learning [16]. It has also been proposed that when a deep learning model performs differently across identical training runs (i.e., identical data, network architecture, software and hardware) there are inherent concerns as to the fairness of the reported performance metrics [17]. Similar to the above literature manuscripts focused on deep learning applications, this manuscript is also concerned with the reproducibility of artificial intelligence technologies; however, the focus is on traditional statistical learning algorithms and the consistency of sample-wise errors across trained models. 

The purpose of this study was to assess whether the variability in the sample-wise consistency of errors (our proposed error consistency metric) made by AI models can act as an informative adjunct to traditional validation approaches, such as K-Fold cross validation. Our objectives were: (1) describe the variability in the sample-wise consistency of errors made by AI models; (2) evaluate proposed error consistency as a measure of AI model reliability across several datasets and learning technologies; and (3) release public domain software to facilitate other researchers in the assessment of error consistency on any given AI classification task. We performed a preliminary analysis of the effects of sample size on error consistency; however, a detailed analysis of sample size effects is beyond the scope of this manuscript and is a subject for future work.

## 2. Materials and Methods

### 2.1. Proposed Error Consistency Validation/Evaluation

The proposed error consistency (EC) evaluative validation method was created as an extension to the existing K-Fold validation strategy [9]. Two main approaches are supported by our validation software, which has been made publicly available [18]. The first approach involves the creation of a single independent validation set on which error consistency is assessed, based on K-Fold validation applied to the training samples. The second approach assesses error consistency internally as part of hold-out K-Fold validation (no separate validation set), by considering the entire training data for calculating predictive error consistency. The first approach relies on additional held-out user-specified samples. The second approach makes full use of all samples available for the assessment of error consistency. In both cases, trained models produce error sets—the collection of held-out samples on which predictive errors were made by the learning algorithms. In the first approach, the error sets are established based on any given model’s errors produced on the user-defined hold-out validation set. In the second approach, the overall error sets are established by combining the error set from each of the K models created in a K-Fold validation run, creating a master error set across all training samples provided to the software. In both approaches, K-Fold validation is repeated *m* times, with *m* set to a high number (*m* = 500), in keeping with best practices for the statistical reliability of validation results.

The overall accuracy (OA) is computed for each of *m* runs of K-Fold validation, and the average OA across validation runs is computed as a standard evaluative metric. In the first approach, each of the *m* runs of K-Fold validation produce K sets of predictions, and thus *n = m* × K corresponding error sets. In the second approach, each of the *m* runs of K-Fold validation produce a single unified set of predictions across all available samples, thus producing *n = m* error sets. The error sets are denoted as *E_i_* or *E_j_* (where *i* or *j* is [1 … *n*]). The error consistency between any two error sets *E_i_* and *E_j_* is defined as the size of the intersection of the two sets divided by the size of the union of the two sets:

(1) $${EC}_{i,j}=size(E_{i}\cap E_{j})/size(E_{i}\cup E_{j})$$

This produces a matrix of error consistency values. *EC_i,i_* will always be 1, as the mistakes of one model will perfectly agree with itself; thus, the diagonal of the error consistency matrix is not relevant for further analysis. Additionally, since *EC_i,j_* is identical to *EC_j,i_*, half the non-diagonal matrix is redundant. As such, in practice, we only compute the *n* × (*n* − 1)/2 half of the non-diagonal entries in the error consistency matrix (i.e., the upper triangular matrix that excludes the diagonal terms). The error consistency matrix can be inspected by the ML application developer to assess the variability of error consistency between model set pairs, and to identify groups of models that behave either particularly similarly or particularly differently from each other. We also summarize with the average (AEC—average error consistency) and the standard deviation (SD) of the upper triangular non-diagonal portion of the error consistency matrix (e.g., all *n* × (*n* − 1)/2 pairings). Note that if the two error sets are empty, we have a pair of perfect classifiers with 100% accuracy, and the size of both the intersection and the union (denominator in Equation (1)) of the two error sets is zero; thus, the error consistency is not a number (NaN), due to the fact that there are no errors upon which to be consistent. This is a rare circumstance in real-world datasets and did not occur in any of the experiments presented in this manuscript.

The code for the additional analyses [19] associated with our public domain error consistency release software [18] also includes plotting routines that are potentially helpful in the design of reliable and consistent machine learning models, and includes the effect of the sample size variance plot. The effect of the sample size variance plot involves randomly down-sampling to a range of proportions of the samples available, performing EC-enhanced K-Fold validation and reporting a scatter plot summarizing the effect of sample size on OA and AEC. A plot whose profile for OA or AEC increases on the right side implies that adding more samples is needed to optimize the performance of the AI model for either metric. 

### 2.2. Biomedical Datasets and Machine Learning Techniques Evaluated

The following datasets were used for this analysis. The number of samples in the dataset is listed (*n*) alongside the number of features (*f*). Three datasets were accessed from the University of California at Irvine Machine Learning Repository (https://archive.ics.uci.edu/ml/index.php, 7 October 2021): Parkinsons (*n* = 195, *f* = 22), SPECT (*n* = 267, *f* = 22), Transfusion (*n* = 748, *f* = 4). The Parkinsons dataset involves diagnosing whether the patient has Parkinson’s disease. The SPECT dataset involves diagnosing normal or abnormal pathology. The Transfusion dataset is an example problem predicting whether a patient would go on to donate blood. The diabetes dataset [20] (*n* = 768, *f* = 8) involves diagnosing whether the patient has diabetes (accessed 7 October 2021). The Heart Failure dataset (https://physionet.org/content/heart-failure-zigong/1.3/, accessed 13 February 2023) was included with 148 features and included 2008 subjects. Heart Failure is a representative prognostic application predicting whether a patient will be re-admitted within six months of the original admission. To aid in reducing computing times on the larger datasets, UMAP [21] was used to reduce categorical features to a manageable number of dimensions. Details are available in the source code. The Diabetes 130 dataset (https://www.openml.org/search?type=data&status=active&sort=runs&id=43903, accessed 13 February 2023) was included with 35 categorical variables summarized as 5 continuous variables, and included 101,766 samples. The diabetes 130 dataset is a representative prognostic application predicting whether a subject is re-admitted 30 days following initial admission. The UTI Antimicrobial Resistance dataset (https://physionet.org/content/antimicrobial-resistance-uti/1.0.0/, accessed 13 February 2023) was included with 713 categorical features summarized as 100 continuous features, and included 100,769 subjects. This diagnostic application involves predicting the existence of any form of antibiotic resistance in the patient. The MIMIC-IV Emergency Visits dataset (https://physionet.org/content/mimic-iv-ed/2.2/, accessed 13 February 2023) was included with 40 categorical features summarized as 5 continuous features and included 369,618 visits from 184,577 unique patients. This application involves predicting patient outcomes. Each dataset was subjected to the second validation approach (internal K-Fold EC, K = 5, *m* = 500), as no secondary independent datasets were available in these applications. Each dataset was compared with the support vector machine (SVM) [22] with a radial basis function kernel, logistic regression, a random forest [23,24] (RF) with 100 decision trees, and AdaBoost (with Decision Tree base learners), all implemented using tools from Python Scikit-learn. Optimal hyperparameters were found for all models using a random search with 5-fold cross-validation on each full dataset.

## 3. Results

**Figure 1:**
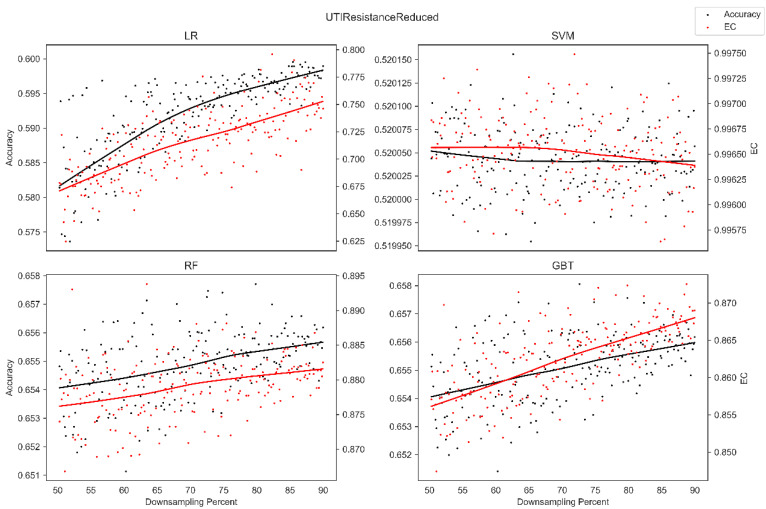
Average error consistency and overall accuracy vs. varying sample size percentages (down-sampled) from the UTI Resistance dataset. Trend lines were established with locally weighted regression and smoothing [25].

**Figure 2:**
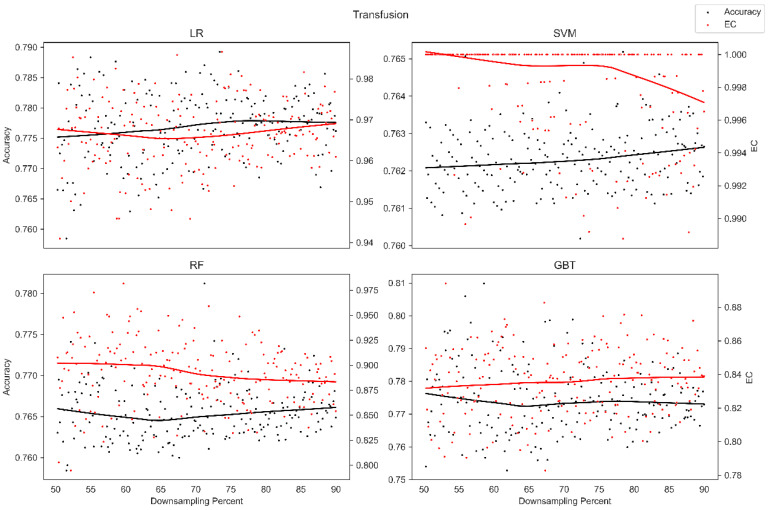
Average error consistency and overall accuracy vs. varying sample size percentages (down-sampled) from the Transfusion dataset. Trend lines were established with locally weighted regression and smoothing [25].

**Figure 3:**
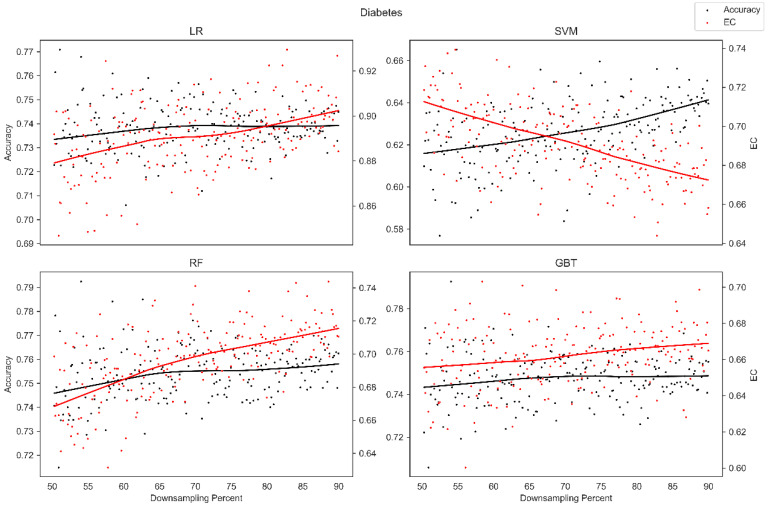
Average error consistency and overall accuracy vs. varying sample size percentages (down-sampled) from the Diabetes dataset. Trend lines were established with locally weighted regression and smoothing [25].

**Figure 4:**
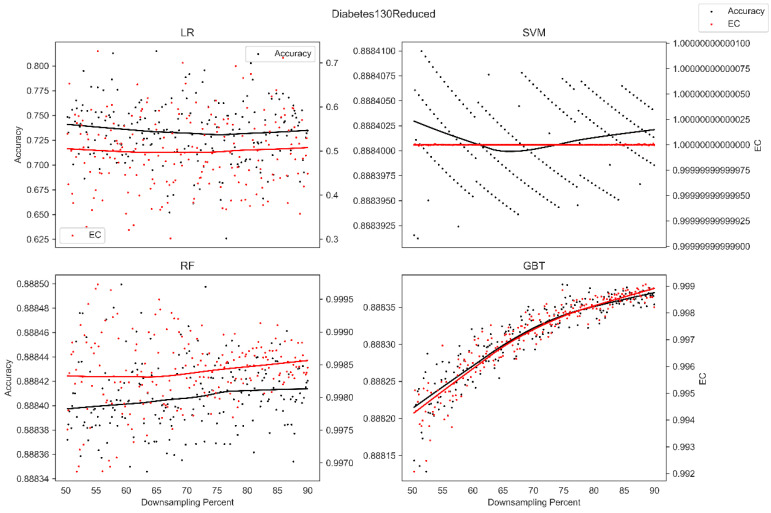
Average error consistency and overall accuracy vs. varying sample size percentages (down-sampled) from the Diabetes 130 dataset. Trend lines were established with locally weighted regression and smoothing [25].

**Figure 5:**
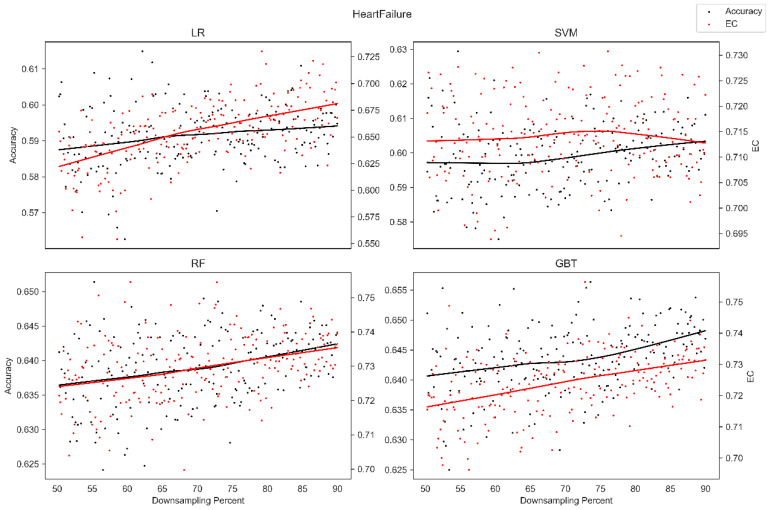
Average error consistency and overall accuracy vs. varying sample size percentages (down-sampled) from the Heart Failure dataset. Trend lines were established with locally weighted regression and smoothing [25].

**Figure 6:**
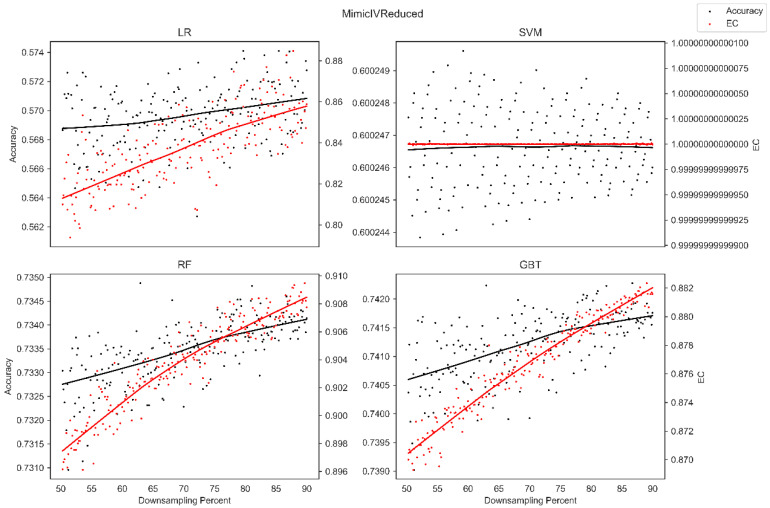
Average error consistency and overall accuracy vs. varying sample size percentages (down-sampled) from the MIMIC IV dataset. Trend lines were established with locally weighted regression and smoothing [25].

**Figure 7:**
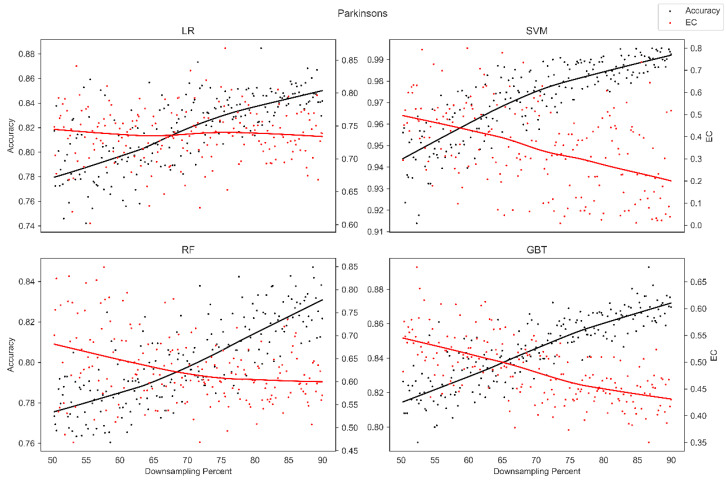
Average error consistency and overall accuracy vs. varying sample size percentages (down-sampled) from the Parkinsons dataset. Trend lines were established with locally weighted regression and smoothing [25].

**Figure 8:**
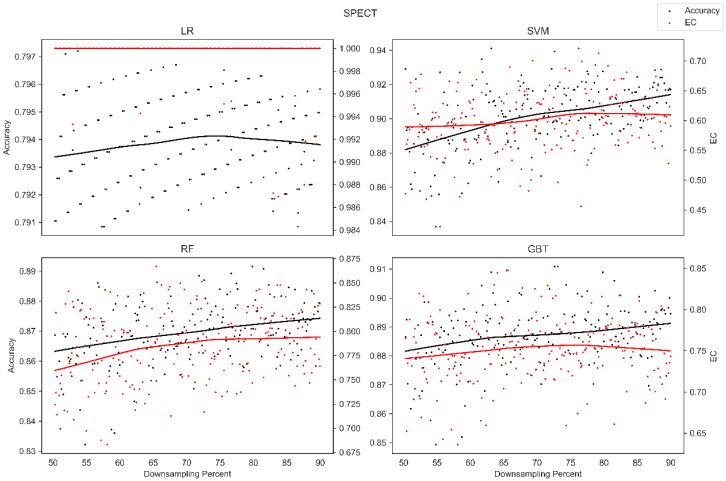
Average error consistency and overall accuracy vs. varying sample size percentages (down-sampled) from the SPECT dataset. Trend lines were established with locally weighted regression and smoothing [25].

The average overall accuracy (OA) as a percentage (%) and its associated standard deviation (SD), as well as the average error consistency (AEC) as a percentage (%) and its associated standard deviation (SD) across validation runs, are presented in Table 1 for all four ML techniques considered across each of the complete (not down-sampled) datasets included in our analysis. Table 1 allows easy comparison of OA and AEC between standard ML algorithms across all datasets considered. Table 1 demonstrates that the random forest and the boosted decision tree methods tend to produce lower error consistency than more traditional statistical-based learning methods such as the support vector machine and logistic regression. Table 1 also demonstrates that error consistency (EC) is dataset-dependent, with varying levels of EC for the same learning technique across datasets. Figure 1, Figure 2, Figure 3, Figure 4, Figure 5, Figure 6, Figure 7 and Figure 8 provide scatter plots of the variability in sample size (through random down sampling) vs. AEC and OA for each of the four ML techniques assessed, providing a visual example of the effect of sample size considerations on both error consistency and accuracy. Figure 1, Figure 2, Figure 3, Figure 4, Figure 5, Figure 6, Figure 7 and Figure 8 were created by randomly down-sampling each dataset and then performing our proposed error consistency (EC)-enhanced K-Fold cross-validation, thus providing information on how error consistency varies with sample size for each of the four ML techniques analyzed. The OA results are also produced by our public domain software package and represent the results of standard K-Fold cross validation for comparison and simultaneous evaluation. Figure 1, Figure 2, Figure 3, Figure 4, Figure 5, Figure 6, Figure 7 and Figure 8 demonstrate a wide variety of possible trends one might observe when applying our software package, in order to assess OA and EC as a function of sample size down-sampling percentage. Locally weighted regression and smoothing [25] was used to establish trend lines across the plots, and to help visually analyze the trajectory of OA and EC as the sample size approaches 100% on the right side of the plots. OA (in black) has a tendency to either plateau or be consistently rising on the right side of the plot, in line with expectation, as it is well known that classifiers tend to improve their predictive accuracy with a larger sample size. EC (in red) is quite variable in the profile of the trend line as the sample size approaches 100% on the right side of the plots. A rising EC trend on the right side of the plot implies that adding more samples will result in more consistency in the sample-wise errors made by the trained models; a plateau on the right side of the plot implies that adding more samples might not change the EC profile of the learned models. Finally, a descending EC trend on the right side of the plot implies that EC will degrade as more samples are added, an effect that is most common when the OA is very high—a situation where there are relatively few total sample-wise errors on which to assess EC. Figures S1–S8 provide bootstrapped rolling window correlations between OA and sample size percentage, as well as between EC and sample size percentage. The figures were created based on 9000 pairwise accuracies and 9000 pairwise ECs, with varying rolling window sizes outlined in columns 2 to 4 from each figure. The leftmost plot of each row in Figures S1–S8 provides all the raw pairwise OAs and ECs. 

## 4. Discussion

An approach to AI model validation and evaluation is presented that extends standard K-Fold validation to the assessment of variability in the sample-wise distribution of erroneous predictions made by the AI models subjected to validation. The software created was made publicly available to facilitate other researchers in assessing the consistency of the errors made by models they are tasked with validating [18].

When assessing the eight datasets included in our analysis, typically, the overall accuracy (OA) clearly rises on the right side of the down-sampling plots (see Figure 1, Figure 2, Figure 3, Figure 4, Figure 5, Figure 6, Figure 7 and Figure 8), implying that adding additional samples will improve classifier accuracy, which is expected, as improved classifier performance with a larger sample size is a common feature of machine learning applications. However, in some situations, the OA approaches a plateau-like pattern at the right side of the down-sampling plots, as we approach the inclusion of all of the samples available (for examples, see Figure 3 upper left and Figure 3 lower right). This implies that adding additional samples may not result in OA performance improvements in these example applications. However, it should be noted that the average error consistency profile clearly increases on the right side of these plots, implying that the accumulation of more samples can help with the creation of more reliable and consistent machine learning models in this application. This is noteworthy, as the findings imply that it is possible to achieve improvements in AI model consistency even when OA improvements might not be obtainable through increased sample size. It is anticipated that this plotting technique can be used to assess whether further performance improvements (in terms of either OA or AEC) are obtainable through further increases in sample size; therefore, this design tool can potentially assist in the creation of more reliable and consistent AI models, and thus, more reliable and consistent AI-based diagnostic applications.

When a series of AI models have been created as part of an in-lab pre-deployment validation procedure, such as K-Fold cross validation, which one should be deployed as the ‘validated’ model? If all the models created share an identical error profile (the collection of samples upon which predictions are incorrect), then it probably will not matter which model from validation is deployed in the real world. However, when the models disagree on the mistakes that they make, it is not clear which one should be deployed, as this model selection choice will affect the mistakes that the deployed technology makes in its real-world application setting. Fortunately, the tool presented in this analysis supports the assessment of the consistency of mistakes across models created as part of in-lab validation, which can be used to help AI application designers with the remarkably challenging task of validation model selection and deployment. It is interesting to note from Table 1, that, for example, in the MIMIC IV dataset, the random forest and AdaBoost produce the highest average overall accuracies of the classifiers assessed; however, the highest average error consistency was obtained with the support vector machine and logistic regression (98%), whereas the random forest and AdaBoost only achieved an error consistency of 91%. This implies that although logistic regression slightly underperforms the random forest in terms of overall accuracy (71% vs. 73%), it is much more consistent across validation in the sample-wise mistakes made by the logistic regression models, implying a level of reliability and predictability in behavior that the random forest might not be capable of. Systemic biases in various AI technologies can have an impact on the assessed ML error consistency behavior. For instance, a random forest, whose training procedure consists of extensive randomization (both of samples and of features), produces high-accuracy models with a tendency for lower error consistency. The extensive randomization in training appears to directly bias the model towards lower error consistency values. This is in stark contrast to more traditional techniques, such as logistic regression, as well as the support vector machine, whose trainer typically uses a quadratic optimizer to select the sample-based support vectors themselves. Thus, although the SVM uses an optimization procedure and minimizes error on unseen samples (targeting a low error rate or high accuracy), the sample-wise errors have a strong tendency to be the same sets of samples located in data space close to the decision boundary established by the SVM classifier. As such, in some situations, the SVM can produce higher error consistency values. In situations where the learning machine achieves nearly perfect error consistency (such as all models in the Diabetes 130 dataset, see Table 1), the deployment of any of the models assessed as part of in-lab validation should produce deployed technologies that are predictable in terms of the types of sample-wise mistakes the AI will make. When traditional metrics for the assessment of model performance in validation (such as OA) are very close to each other, then perhaps it is the techniques that are more consistent in their error profiles that would be easiest to deploy in real-world applications without introducing ambiguity with respect to how it will behave (and on what sample types it will fail) after deployment. However, when AI model developers have a series of lab-validated technologies to select between for real-world deployment, how would one choose between models with higher OA and lower EC or vice versa? This is a fundamentally application-specific design consideration, whereby either the models with higher OA, or those with higher predictability and reliability (as assessed with EC), may be the most appropriate deployment technology given the accuracy and reliability/predictability demands of the application at hand. The tool presented in this manuscript supports AI application developers to assess the reliability and predictability of mistakes made by their models alongside model OA, supporting the developer to make informed decisions regarding model deployment.

In mission-critical applications such as medical diagnostics, the importance of being able to explain what the ML model has learnt is particularly important. For example, in medical applications, detailed knowledge of the abnormal presentation of a pathology can help clinicians and researchers better understand the conditions they manage, and may help inspire new treatments targeting regions not previously known to have been involved in the given medical condition. The importance of being able to convey what ML models have learnt is paramount in mission-critical applications, so it is particularly undesirable for ML models created as part of standard validation to disagree with each other in major parts of their functionality. ML application developers already have a major challenge associated with explaining what their ML models have learnt, and thus, what their technology is relying upon to make its predictions, especially in clinical diagnostics. When validation produces many models, each of which learnt something different from one another, explaining what the models have learnt becomes a much more challenging task. Focusing only on explaining what the deployed model has learnt results in concerns about the underlying validation employed, if in-lab-assessed model performance was averaged across many models, each of which learnt something quite different from one another.

Two approaches to our proposed enhanced validation are supported in the software: the first involves the user defining a validation set upon which error consistency is evaluated with K-Fold cross validation employed on the training set, and the second approach involves assessing error consistency internally as part of traditional K-Fold validation on the internally held-out testing sets. The first approach, which employs a user-defined held-out validation set, is provided for situations where an independent validation set is available to the user and situations where excessive numbers of samples are available, supporting the partition of datasets into training and validation sets of sufficiently large size. The second approach, which estimates error consistency internally as part of K-Fold validation, was developed to support the assessment of error consistency in situations where only a single dataset is available and for handling datasets of small sample size. Since the datasets accessed do not have independent corresponding datasets, the second approach, whereby error consistency is assessed internally as part of K-Fold cross validation, was selected for the main findings in this manuscript. Although we provided sample size down-sampling plots (see Figure 1, Figure 2, Figure 3, Figure 4, Figure 5, Figure 6, Figure 7 and Figure 8) and rolling window correlation plots (see Figures S1–S8) for eight datasets, this represents a preliminary assessment of sample size considerations. Future work will investigate sample size effects in extremely large-scale datasets in detail.

Future work will investigate sample size considerations in convolutional neural networks (CNNs), error consistency in CNNs and whether error consistency metrics can be useful for the development of ensembles of deep learning techniques. CNNs are very flexible in their architectural structure definition and are seeded by randomly initialized weights; as such, it is difficult to predict the error consistency of a given deep learning network for a given learning application. Fortunately, the software developed as part of this study is compatible with the assessment of error consistency in CNNs, and so it can be used to inform design considerations in deep learning. Ensembles of CNNs have demonstrated considerable potential towards their application in bioinformatics learning challenges [26]. Typically, base learner CNNs are aggregated at the ensemble layer with a simple vote, and it is known among researchers that forcing the underlying models to be different from one another, and thus, learn different things from the dataset, is advantageous to the performance gain observed from an aggregated vote [27]. With the development of error consistency metrics, we have a method for assessing the potential for an aggregated vote to improve ensemble performance over that of its base learners. Perfect error consistency will correspond to no improvement in an aggregated vote, whereas the lower the error consistency, the more improvement from ensemble voting is expected. Although we investigated four classifiers on eight datasets, the technique developed is compatible with any classifier and any dataset, as long as the task at hand is classification-based supervised learning. It is encouraging that a small number of studies have emerged recently considering issues related to consistency in deep learning [15,16,17,28,29,30,31,32], technologies that include enormous numbers of randomly initialized parameters and so are prone to producing inconsistent solutions when trained repeatedly. Future work will investigate the application of error consistency to deep learning in detail (as was done in this statistical learning study), alongside the development of training-integrated techniques to assess model error consistency as training epochs unfold.

In conclusion, our findings imply that many factors affect error consistency, including the machine learning technology relied upon, the distribution of data in the dataset available, as well as sample size issues. It is also anticipated that additional factors may affect error consistency, including underlying feature quality. Error consistency is intended to act as an adjunct alongside traditional metrics such as overall accuracy. When a machine learning technology is either 100% accurate or 100% inaccurate, error consistency is irrelevant, as either all samples are predicted correctly or incorrectly, respectively. However, in most real-world situations, a learning technology will not exhibit perfect performance, and so assessing a technology’s error consistency may be valuable for model evaluation and validation. Unfortunately, it is impossible to know in advance how error-consistent a given machine learning technology will be when subjected to validation on a given dataset/application; however, with the software tools presented in this manuscript, any machine learning application developer can now assess their supervised classification model’s in-lab validated error consistency using our standardized public domain tool. We are hopeful that this tool will support the development and deployment of reliable and predictable medical diagnostic applications of AI.

## Figures and Tables

**Table 1:** Average overall accuracy (SD)/average error consistency (SD) for each ML model and dataset combination considered. All entries in the table report percentages. Sample sizes are provided in Section 2.2.

Dataset	Support Vector Machine	Random Forest	Logistic Regression	AdaBoost DT
Diabetes	75 (0.6)/72 (2.5)	76 (0.5)/72 (3.0)	77 (0.3)/90 (2.1)	76 (0.6)/68 (2.7)
Diabetes 130	89 (0.0)/100 (0.0)	89 (0.0)/100 (0.0)	89 (0.0)/100 (0.0)	89 (0.0)/100 (0.0)
Heart Failure	64 (0.4)/75 (1.3)	64 (0.4)/75 (1.4)	62 (0.4)/79 (1.2)	65 (0.4)/75 (1.2)
MIMIC IV	60 (0.1)/98 (1.1)	73 (0.01)/91 (0.2)	71 (0.0)/98 (0.2)	74 (0.0)/89 (0.1)
Parkinsons	88 (0.5)/88 (5.5)	84 (1.2)/70 (11.5)	85 (0.5)/90 (4.8)	87 (1.2)/49 (7.8)
SPECT	83 (0.8)/74 (5.2)	83 (0.5)/87 (4.9)	84 (0.7)/82 (5.2)	84 (0.6)/80 (5.0)
Transfusion	76 (0.2)/93 (1.7)	76 (0.2)/96 (4.0)	77 (0.2)/96 (1.4)	77 (0.4)/86 (2.3)
UTI Resistance	51 (1.1)/38 (3.3)	66 (0.0)/88 (0.2)	64 (0.0)/92 (0.7)	66 (0.0)/87 (0.2)

## Data Availability

All data is publicly available, see citations and links in the manuscript.

## Supplementary Materials

The following supporting information can be downloaded at: https://www.mdpi.com/article/10.3390/diagnostics13071315/s1, Figure S1. Correlations between Average Error Consistency and downsampling percentage, as well as Overall Accuracy and downsampling percentage from the UTI Resistance dataset. Trend lines were established with locally weighted regression and smoothing [25]; Figure S2. Correlations between Average Error Consistency and downsampling percentage, as well as Overall Accuracy and downsampling percentage from the Transfusion dataset. Trend lines were established with locally weighted regression and smoothing [25]; Figure S3. Correlations between Average Error Consistency and downsampling percentage, as well as Overall Accuracy and downsampling percentage from the Diabetes dataset. Trend lines were established with locally weighted regression and smoothing [25]; Figure S4. Correlations between Average Error Consistency and downsampling percentage, as well as Overall Accuracy and downsampling percentage from the Diabetes 130 dataset. Trend lines were established with locally weighted regression and smoothing [25]; Figure S5. Correlations between Average Error Consistency and downsampling percentage, as well as Overall Accuracy and downsampling percentage from the Heart Failure dataset. Trend lines were established with locally weighted regression and smoothing [25]; Figure S6. Correlations between Average Error Consistency and downsampling percentage, as well as Overall Accuracy and downsampling percentage from the MIMIC IV dataset. Trend lines were established with locally weighted regression and smoothing [25]; Figure S7. Correlations between Average Error Consistency and downsampling percentage, as well as Overall Accuracy and downsampling percentage from the Parkinsons dataset. Trend lines were established with locally weighted regression and smoothing [25]; Figure S8. Correlations between Average Error Consistency and downsampling percentage, as well as Overall Accuracy and downsampling percentage from the SPECT dataset. Trend lines were established with locally weighted regression and smoothing [25].
