# Supplementary material for: Error Consistency for Machine Learning Evaluation and Validation with Application to Biomedical Diagnostics
Source: Diagnostics (Basel). 2023 Apr 1;13(7):1315. doi: 10.3390/diagnostics13071315 (PMC10093437; doi:10.3390/diagnostics13071315)
Supplement: Supplementary file 1 [file diagnostics-13-01315-s001.zip › diagnostics-2199745-supplementary.pdf]

Supplementary Materials:

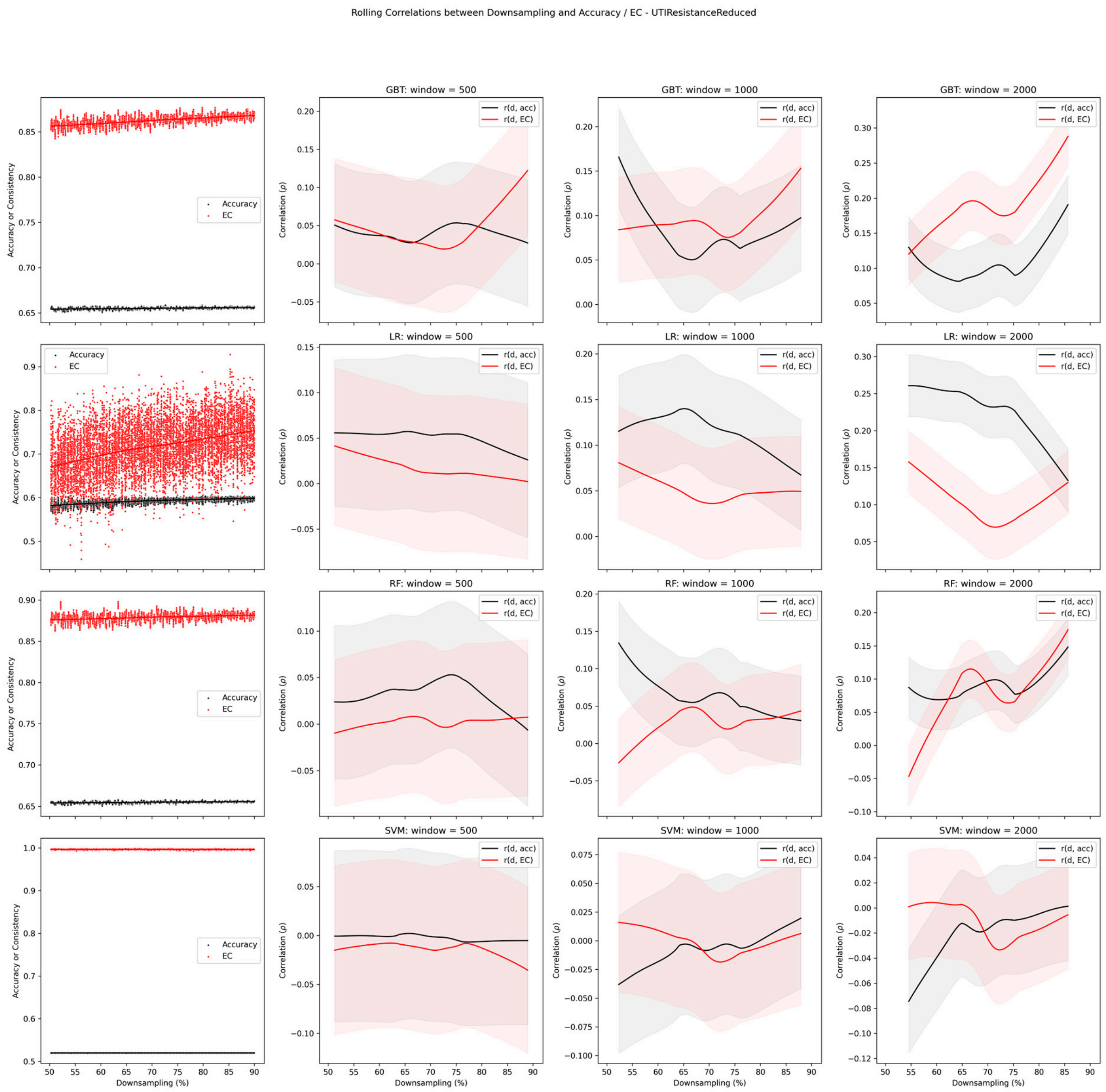

Figure S1. Correlations between Average Error Consistency and downsampling percentage, as well as Overall Accuracy and downsampling percentage from the UTI Resistance dataset. Trend lines were established with locally weighted regression and smoothing [25].

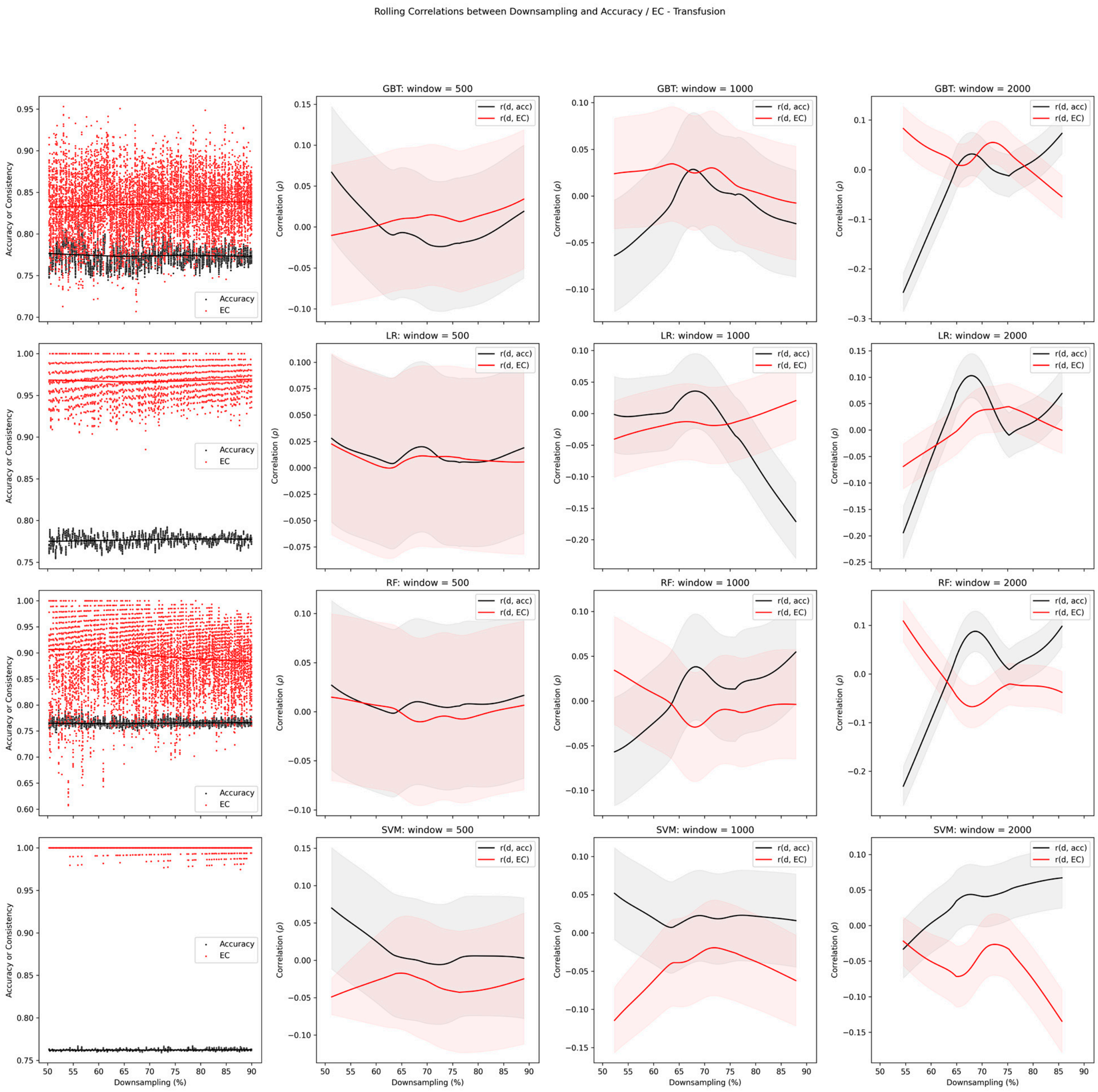

Figure S2. Correlations between Average Error Consistency and downsampling percentage, as well as Overall Accuracy and downsampling percentage from the Transfusion dataset. Trend lines were established with locally weighted regression and smoothing [25].

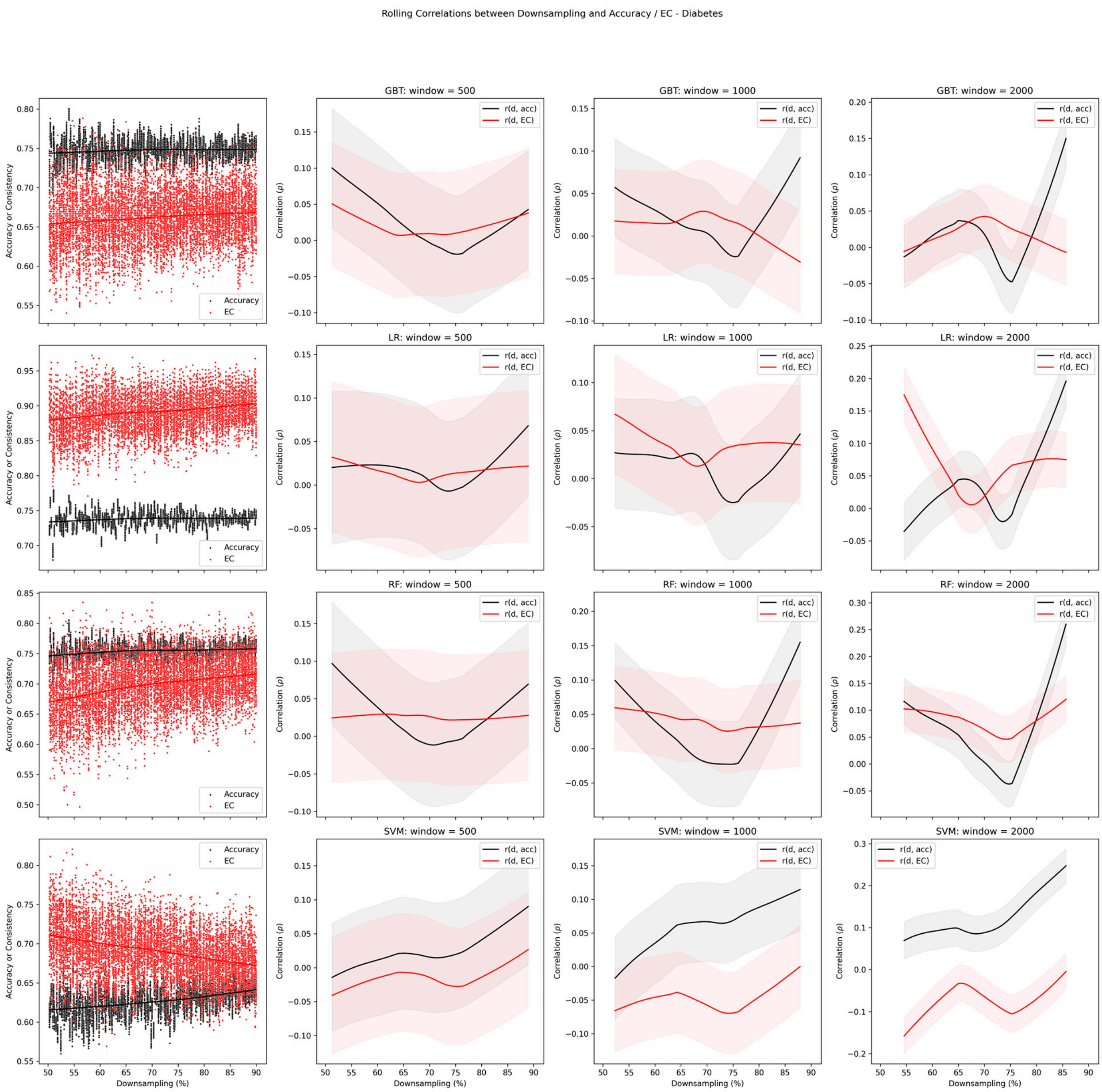

Figure S3. Correlations between Average Error Consistency and downsampling percentage, as well as Overall Accuracy and downsampling percentage from the Diabetes dataset. Trend lines were established with locally weighted regression and smoothing [25].

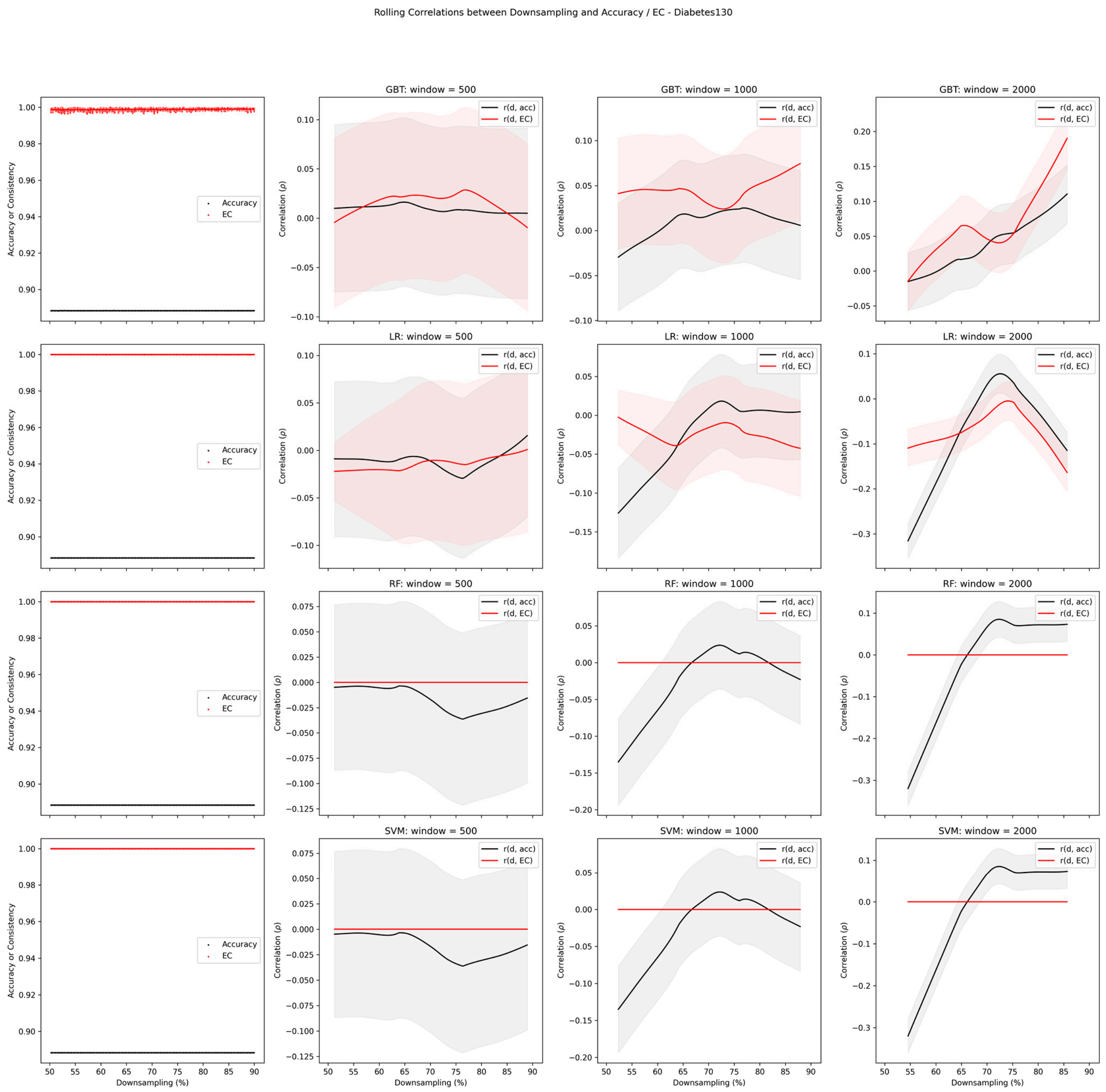

Figure S4. Correlations between Average Error Consistency and downsampling percentage, as well as Overall Accuracy and downsampling percentage from the Diabetes 130 dataset. Trend lines were established with locally weighted regression and smoothing [25].

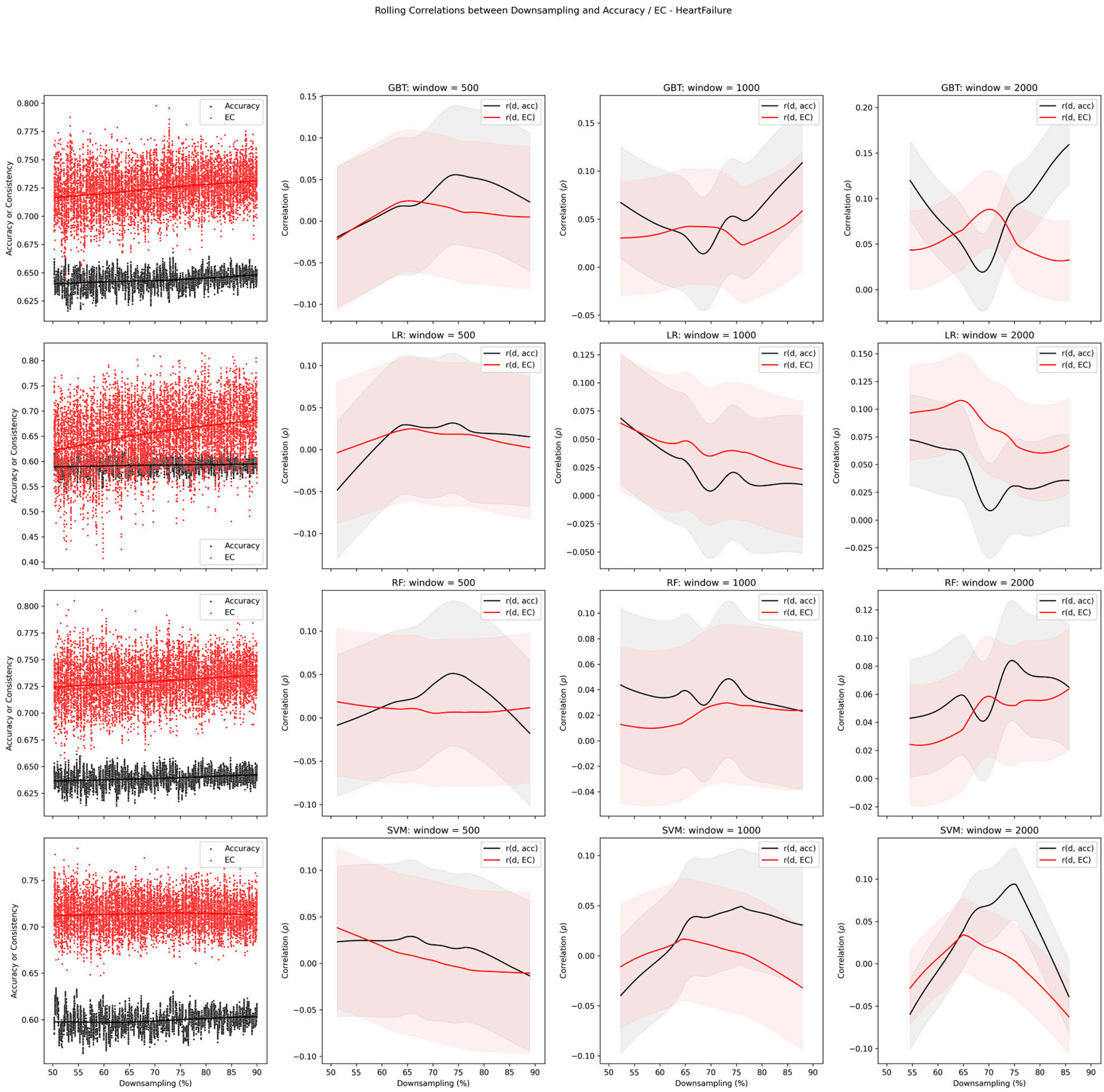

Figure S5. Correlations between Average Error Consistency and downsampling percentage, as well as Overall Accuracy and downsampling percentage from the Heart Failure dataset. Trend lines were established with locally weighted regression and smoothing [25].

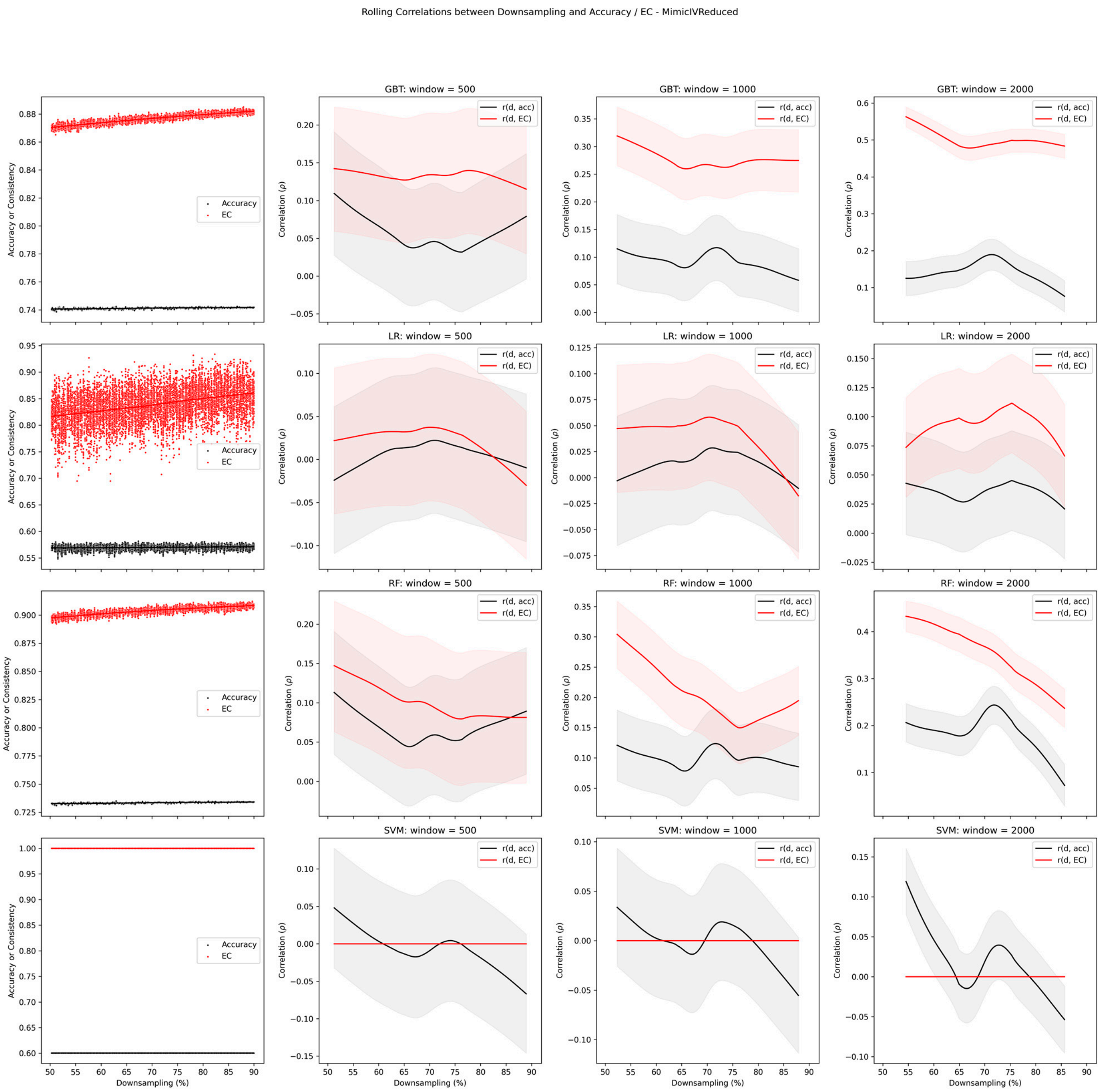

Figure S6. Correlations between Average Error Consistency and downsampling percentage, as well as Overall Accuracy and downsampling percentage from the MIMIC IV dataset. Trend lines were established with locally weighted regression and smoothing [25].

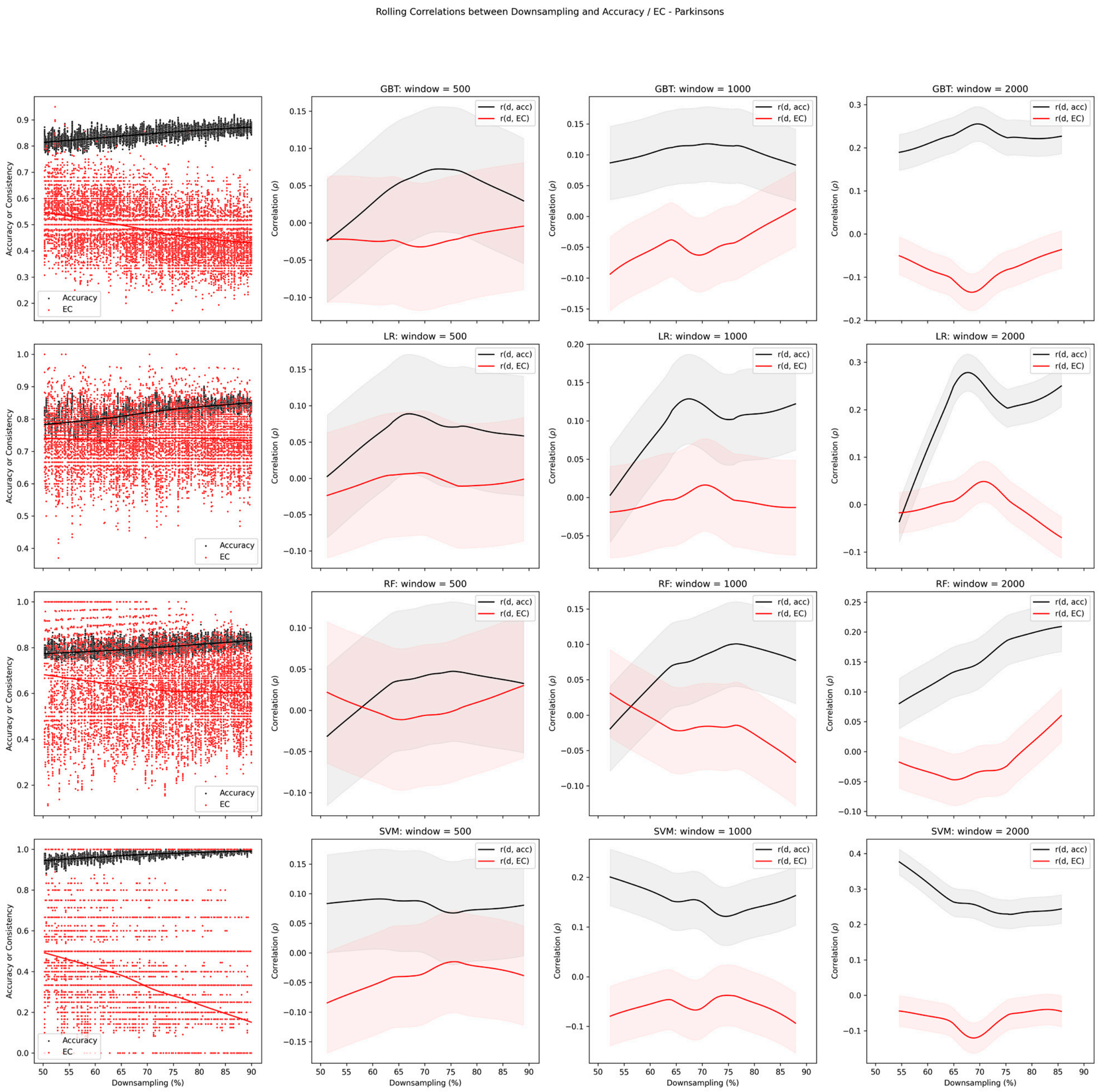

Figure S7. Correlations between Average Error Consistency and downsampling percentage, as well as Overall Accuracy and downsampling percentage from the Parkinsons dataset. Trend lines were established with locally weighted regression and smoothing [25].

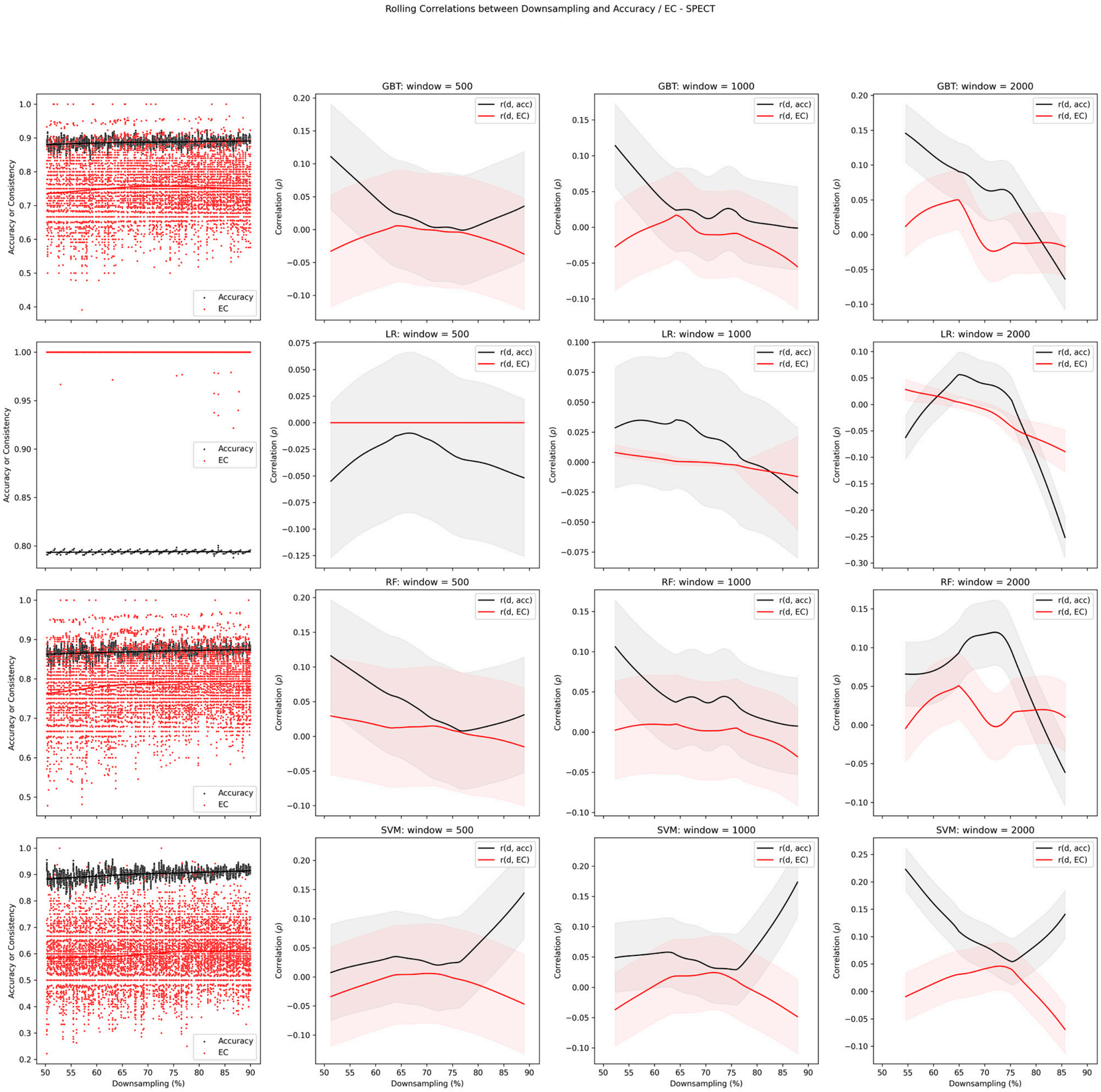

Figure S8. Correlations between Average Error Consistency and downsampling percentage, as well as Overall Accuracy and downsampling percentage from the SPECT dataset. Trend lines were established with locally weighted regression and smoothing [25].
